# Effects of a mental health promotion intervention on mental health of Iranian female adolescents: a school-based study

**DOI:** 10.1186/s13034-020-00342-6

**Published:** 2020-09-22

**Authors:** Haleh Heizomi, Hamid Allahverdipour, Mohammad Asghari Jafarabadi, Devender Bhalla, Haidar Nadrian

**Affiliations:** 1 Department of Health Education and Promotion, Faculty of Health, Tabriz University of Medical Sciences, Attar-e-Neyshabouri St., Tabriz, Iran; 2 Research Center of Psychiatry and Behavioral Sciences, Tabriz University of Medical Sciences, Tabriz, Iran; 3 Road Traffic Injury Research Center, Tabriz University of Medical Sciences, Tabriz, Iran; 4 Department of Statistics and Epidemiology, Faculty of Health, Tabriz University of Medical Sciences, Tabriz, Iran; 5 Sudan League of Epilepsy and Neurology (SLeN)®, Khartoum, Sudan; 6 Iranian Epilepsy Association®, Tehran, Iran; 7 Nepal Interest Group of Epilepsy and Neurology (NiGEN), Kathmandu, Nepal; 8 Social Determinants of Health Research Center, Tabriz University of Medical Sciences, Attar-e-Neyshabouri St., Tabriz, Iran

**Keywords:** Mental health, Adolescents, Happiness, Stress management, Mental health promotion

## Abstract

**Background:**

Poor mental health is common among adolescents. Given the increasing burden of poor mental health among adolescents in developing countries, it seems necessary to identify the effective interventions. The main purpose of this study was to investigate the effects of a school-based mental health promotion program (SMHPP) on mental health parameters among female adolescents in Tabriz, Iran.

**Method:**

In this experimental study, a random sample of female high-school students of grade nine was recruited. The subjects were then randomly allocated to intervention (n = 145) and control (n = 139) groups. The three-stage SMHPP was designed based on the shortages and unmet needs of the students as reported in the pretest stage. All subjects in the intervention group were provided with a stress management skill training program of six sessions using McNamara Model. Coincided with making environmental changes, a joyful intervention program was carried out. After 2 months, post-test data were collected.

**Results:**

A total of 284 students completed their participation in the study. The groups did not differ in none of socio-demographic characteristics and mental health parameters, at baseline. The number of subjects reporting medium-level of happiness was increased by 32.6% among intervention group. Moreover, upon sign test and pre-post comparison, the group-wise distribution changed between the intervention and control groups for the parameters of life satisfaction (p ≤ 0.001) and psychological well-being (p ≤ 0.01).

**Conclusion:**

The implementation of SMHPP as a low-cost, needs-based and multifaceted program, showed promise in promoting adolescents’ mental health, particularly in the parameters of happiness, life satisfaction and psychological well-being. This was an important evidence for the development and implementation of interventions and policies in the field of mental health promotion among adolescents. Our work provided means for reducing burden of poor mental health among adolescents in a non-western cultural context. Further larger studies are required to evaluate the effectiveness of such school-based mental health promotion interventions in students.

## Background

As a complex concept, mental health goes beyond the mere absence of mental illness, and is a collaborative state of physical, mental, spiritual, and social well-being [1, 2]. One of the pressing risk groups for poor mental health is adolescents [3], especially school-based ones [4, 5] and females [6], who have shown a dramatic rise in their poor mental health burden in the last few decades [7, 8]. One in eight adolescents experiences a mental disorder, and about one in five youth experiences some forms of developmental, emotional and behavioral problems [9].

Problematic school behaviors have been shown to be affected by excessive and/or prolonged psychosocial stress, a challenging and potentially malleable factor in adolescents [6]. It is also demonstrated that negative emotional responses in stressful situations, such as anxiety, frustration and depression are strongly associated with stress [10]. School-aged children report having numerous stressors in their day-to-day experience, including concerns about poor academic performance, peer exclusion, being bullied or teased and homework [11]. Such stressors may result in lack of concentration, poor performance in decision-making and problem-solving processes, as well as lacks in abilities needed for learning [12].

Several previous studies have shown the role of stress management interventions on promoting acute psychophysiological response to stress in healthy individuals [13], and improving self-esteem/mood [14], decreasing fatigue, anxiety [15] and distress [16] in different populations. Equipping adolescents with coping strategies may help them in better dealing with their stressors and prevent the possible impacts on their future health [17]. Stress management interventions have also been reported to improve the individuals’ control over physical and emotional states, promote confrontation with their stressful situations and improve their health status [18]. Stress management training can also promote happiness [19, 20], which in turn improves mental health status [21]. Training students on how to practice [22] relaxation techniques [23, 24] is also reported as a potentially helpful school-based health promoting intervention. As evident, such interventions have shown promise in preventing mental disorders and promoting mental health among adolescents at schools [25].

Schools provide an ideal setting to promote interpersonal and coping skills [26]. In school mental health promotion programs, if the main approach is focused on mental health promotion rather than mental illness prevention, a better enhancement may be expected at the levels of either mental health or psychological well-being among adolescents [27]. However, mental health promotion may not solely take place through exploring the demographic correlates and predictors of mental health well-being [28]. For instance, stress and self-esteem are strongly associated to physical morbidity [29], and engagement in physical activities leads to lasting mental health improvement [30]. Thus, any genuine approach to improve mental health have to encompass the value of integrating both physical and mental components [28].

Confronted with limited resources and inequities in access to mental health care, developing countries are facing with the impact of mental health problems [1, 3, 31, 32]. About 70.0% of adolescents live in these countries, where complex economic, social, political and environmental contexts may create a wide range of challenges for them to overcome. Many of the disadvantaged adolescents may have few personal resources and little social support to confront with their poor mental health status. Given the increasing burden of poor mental health among adolescents in developing countries [33, 34], it seems necessary to identify effective interventions. In Iran, as a developing country, the picture of mental health is starting to change [35], and more attention is now paid on adolescents and youth, but especially university students [35]. A recent study [36] shows that Iranian adolescents at the middle schools are having trouble with their mental health. Thus, with such vision, our aim in the present study was to determine the effect of a school-based mental health promotion program (SMHPP) on a series of pre-chosen mental health predictors among female high-school students in Iran.

## Methods

### Design, participants and sampling

This intervention was a quasi-experimental field study with control group carried-out among female high school students of grade nine in Tabriz, Iran. Among all five educational districts in Tabriz, we randomly selected one district–district number four with three sub-districts. Next, in the selected district, we again randomly selected two high schools with almost the same educational and environmental characteristics, as the intervention and control groups. The grade nine students at the selected schools (148 in intervention and 141 in control groups) were all invited to participate in the study. So, a total of 289 students were included of which 284 subjects (145 in intervention and 139 in control groups) completed their participation in the study. Two subjects in the control group refused to participate in the study, and three subjects in the intervention group did not complete at least 80% of the intervention sessions (participation rate = 98.2%). The educational and environmental characteristics included the overall socio-economic status of the locations where the schools selected, fields and grades covered by the schools, quality and quantity of the school’s buildings (recreational and sport places, building age, green space, etc.), number of students in each class and the number of staff at the schools. Thereafter, the participants were invited based on the following criteria: with regular attendance at schools, no prior history of mental health intervention in the past 3 years, not using/abusing substances and able and willing to participate, independently. The implementation of program lasted for 2 months. The intervention and control groups were assessed at two study time points (before and 2 months after the intervention). Participants who did not participate regularly in at least 80% of the sessions were not included into the post-test data collection section.

The study had three stages. In the first stage, all students in the intervention group underwent stress management skills training. In the second stage, based on the findings of the pretest data analysis [37], environmental changes were made within the school. In the third stage, a joyful program intervention was conducted. Those in the control group (as a comparator) received only the routine mental health measures conducted by the health station situated at the school.

All participants and at least one of their parents were explained about the purpose of the study and were ensured about the confidentiality of their information. They also signed an informed consent form.

### Measures

The background data were collected using a questionnaire consisting demographic information on age, number of family members, and parents’ job and education level. Moreover, applying two questions, economic status (How do you rate the economic status of your family? Answer choices: low/moderate/high) and religious beliefs (How do you describe your religious beliefs? Answer choices: no belief/weak belief/moderate belief/strong belief) were measured. The Persian versions of General Health Questionnaire (GHQ-28) (Cronbach’s alpha = 0.92), Oxford Happiness Questionnaire (Cronbach’s alpha = 0.85), Sherer’s General Self-efficacy Scale (Cronbach’s alpha = 0.81), Cohen’s Perceived Stress Scale (Cronbach’s alpha = 0.69), Snyder’s Hopefulness Scale (Cronbach’s alpha = 0.53) and Diener’s Satisfaction with Life Scale (SWLS) (Cronbach’s alpha = 0.80) were applied to measure psychological well-being, happiness, self-efficacy, perceived stress, hopefulness and life satisfaction, respectively. More information on the scales and their associated scoring as well as their validity and reliability on this population have been previously described in detail [38].

### Mental Health Promotion Program

#### Stress management skills training program

The training was based on McNamara’s model (Table 1), and lasted six weekly sessions provided by a trained clinical psychologist through lectures, practice and questions/answers sessions. At the end of each training session, important summarized notes taken from the training were given to all participants in the form of leaflets. Each session lasted about 45 to 60 min.

**Table 1:** Profile of the proposed training program for students

Meetings	Topic of meetings
Session 1	Providing an introduction to the importance and necessity of stress control skills and enhancement of mental health Defining stress and dealing with individual differences in the face of stress
Session 2	Introducing the overall impact of stress on different body systems and evaluating the effects of physical, psychological, and behavioral stress
Session 3	Introducing a variety of strategies for coping, problem-focused, and emotion-focused techniques as a way of coping, and comparing healthy and unhealthy solutions
Session 4	Tips for coping with stress and introduction of steps of coping with stress
Session 5	Strengthening confidence and self-esteem and combating depression and anxiety
Session 6	Practical instruction of relaxation techniques, exercising, and repeating it, to ensure the resolution of ambiguities and students’ learning of these skills

#### Environmental changes

The environmental changes were made within the school on the basis of shortages and unmet needs that the participants reported to have in the pre-test data [37]. These changes included bulletin decoration, playing music in break time, counseling and establishing a close relationship between the personnel and the students and holding sports, drawing and cooking competitions.

#### Joyful program intervention

This program was also prepared on the basis of the shortages and unmet needs that the participants reported to have in the pre-test data [37]. The domains considered in the intervention were physical activity, healthy diet, cross-gender social relationship, how to create happy times for oneself and stress (Table 2).

**Table 2:** Key points of the pretest stage for students

The weaknesses of the pre-test stage	Key strategies	Implementation of programs
Little physical activity	Designing programs to increase physical activity	Tug of war competition among students
Not having a healthy diet	Designing programs to encourage teens to eat healthy foods	Holding cooking competition among students
Having a boyfriend	Designing programs for better communication with consultants	Intimate communication with students and answering to their problems
Willingness to create happy times	Designing programs to expose teens to music	Playing music in break time
Having stress	Stress management skill training	Implementation of six sessions of stress management skills based on McNamara’s model through lectures

### Statistical analyses

The data were described in terms of number, frequency, means and standard deviation (SD). The sign test and McNemar’s test were used for within-group comparisons for both intervention and control groups, separately. Between-group comparisons were also done by preparing the cross-tabs for all included mental health predictors according to the distribution of three tertiles of response (low, medium and high) individually. To compare mean score of the psychological wellbeing and its associated factors before and after the intervention, paired *t* test was used. All statistical analyses were performed using the statistical software package SPSS v. 17 (SPSS Inc. IL, Chicago, USA).

## Results

A total of 284 subjects completed their participation in the study. The intervention and control groups did not differ in none of the socio-demographic characteristics (Table 3). The groups had also no statistically significant difference in the mental health parameters, at baseline (Table 4).

**Table 3:** Comparison of background variables in experimental and control groups

Variables	Experimental group N (%)	Control group N (%)	*p* value
Number of family members			
3	27 (27.8)	21 (15.1)	0.09
4	54 (55.7)	95 (68.3)	
≥ 5	16 (16.5)	23 (16.5)	
Father’s job			
Self-employed	62 (64.0)	93 (66.9)	0.40
Employed	35 (36.1)	46 (33.1)	
Mother’s job			
Homemaker	80 (82.4)	114 (82.0)	0.48
Employed	17 (17.5)	25 (18.0)	
Father’s education level			
Elementary school	26 (26.8)	30 (21.5)	0.20
High-school diploma	33 (34.0)	65 (46.8)	
University	38 (39.2)	44 (31.7)	
Mother’s education level			
Elementary school	36 (37.2)	52 (32.4)	0.99
High-school diploma	46 (47.4)	67 (48.2)	
University	15 (15.5)	20 (14.4)	
Economic status			
Low	25 (25.8)	42 (30.2)	0.72
Moderate	63 (64.9)	87 (62.6)	
High	9 (9.3)	10 (7.2)	
Religious beliefs			
No belief	9 (9.3)	17 (12.3)	0.45
Weak	42 (43.3)	60 (43.2)	
Moderate	36 (37.1)	56 (40.3)	
Strong	10 (10.3)	6 (4.3)	

Experimental group (n = 148), Control group (n = 141); Chi Squared Test

**Table 4:** Comparison of major variables in experimental and control groups

Variables	Experimental group M (SD)	Control group M (SD)	p-value
Age	14.67 (0.46)	14.76 (0.42)	0.08
Self-efficacy	58.63 (10.17)	58.09 (10.04)	0.65
Hopefulness	31.76 (3.75)	31.51 (4.08)	0.18
Life satisfaction	20.50 (6.21)	21.02 (6.75)	0.50
Stress	21.26 (5.67)	21.86 (6.05)	0.22
Happiness	1.12 (17.76)	1.13 (21.25)	0.10
Psychological well-being	31.35 (16.33)	30.71 (4.08)	0.96

Experimental group (n = 148), Control group (n = 141); Independent Samples Test

Based on a group-wise comparison between intervention and control groups, there was no statistically significant change in the mental health parameters, except a near significance for medium-level of happiness (OR 1.54, 95% CI 0.91–2.61, p = 0.08). Upon sign test and based on pre-post testing, we found that the group-wise distribution changed between intervention and control groups for the parameters of life satisfaction (p < 0.001) and happiness (p < 0.001). As there is shown in Table 5, the frequency percent of subjects reporting low and average levels of happiness in the intervention group decreased from 32.4 and 39.4% to 24.7% and 29%, respectively. Also, the frequency percent of students reporting high levels of happiness in the intervention group increased from 28.2\ to 46%. Such a difference was also found in life satisfaction (Table 5).

**Table 5:** Major outcome variables before and after intervention in two groups

Variables	Groups	Total^a^	After Intervention, n (%)			p-value*
			Low	Average	High	
Life satisfaction Before intervention	Experimental (n = 145, 100%)					
	Low	52 (36.1)	26 (18.1)	17 (11.8)	9 (6.2)	< 0.001
	Average	51 (35.5)	10 (6.9)	20 (13.8)	21 (14.8)	
	High	42 (28.4)	1 (0.7)	9 (6.3)	32 (21.4)	
	Total^b^	–	37 (25.7)	46 (31.9)	62 (42.4)	
	Control (n = 139, 100%)					
	Low	49 (35.3)	27 (19.4)	8 (5.8)	14 (10.1)	0.22
	Average	39 (28)	12 (8.6)	17 (12.2)	10 (7.2)	
	High	51 (36.7)	1 (0.7)	9 (6.5)	41 (29.5)	
	Total^b^	–	40 (28.7)	34 (24.5)	65 (36.8)	
Happiness Before intervention	Experimental (n = 145, 100%)					
	Low	47 (32.4)	23 (15.8)	14 (9.7)	10 (6.9)	< 0.001
	Average	57 (39.4)	7 (4.8)	20 (13.8)	30 (20.7)	
	High	41 (28.2)	6 (4.1)	8 (5.5)	27 (18.6)	
	Total^b^	–	36 (24.7)	42 (29)	57 (46)	
	Control (n = 139, 100%)					
	Low	48 (53.7)	30 (21.6)	8 (5.8)	10 (7.2)	0.09
	Average	41 (29.5)	9 (6.5)	17 (12.2)	15 (10.8)	
	High	50 (36)	4 (2.9)	7 (5.0)	39 (28.1)	
	Total^b^	–	43 (31)	32 (23)	64 (46.1)	
Self-efficacy Before intervention	Experimental (n = 145, 100%)					
	Low	49 (33.7)	36 (24.7)	9 (6.2)	4 (2.8)	0.59
	Average	56 (38.8)	17 (11.7)	26 (17.8)	13 (9.3)	
	High	40 (27.5)	2 (1.4)	12 (8.3)	26 (17.9)	
	Total^b^	–	55 (37.8)	47 (32.3)	43 (30)	
	Control (n = 139, 100%)					
	Low	55 (39.6)	34 (24.5)	17 (12.2)	4 (2.9)	0.35
	Average	42 (30.1)	12 (8.6)	18 (12.9)	12 (8.6)	
	High	42 (30.3)	4 (2.9)	9 (6.5)	29 (20.9)	
	Total^b^	–	50 (36)	44 (31.6)	45 (32.4)	
Hopefulness Before intervention	Experimental (n = 145, 100%)					
	Low	53 (36.5)	36 (24.8)	7 (4.8)	10 (6.9)	1.00
	Average	44 (31.3)	15 (10.3)	12 (8.3)	17 (11.7)	
	High	48 (33.1)	8 (5.5)	10 (6.9)	30 (20.7)	
	Total^b^	–	59 (40.6)	29 (20)	57 (39.3)	
	Control (n = 139, 100%)					
	Low	49 (35.3)	37 (26.6)	8 (5.8)	4 (2.9)	0.62
	Average	47 (33.9)	19 (13.7)	9 (6.5)	19 (13.7)	
	High	43 (30.9)	6 (4.3)	11 (7.9)	26 (18.7)	
	Total^b^	–	62 (44.6)	28 (20.2)	49 (35.3)	
Stress Before intervention	Experimental (n = 145, 100%)					
	Low	64 (44.1)	45 (31.0)	11 (7.6)	8 (5.5)	0.21
	Average	40 (27.6)	21 (14.5)	4 (2.8)	15 (10.3)	
	High	41 (28.2)	17 (11.7)	8 (5.5)	16 (11.0)	
	Total^b^	–	83 (57.2)	23 (15.9)	39 (26.9)	
	Control (n = 139, 100%)					
	Low	58 (41.7)	41 (29.5)	16 (11.5)	1 (0.7)	0.11
	Average	37 (26.6)	17 (12.2)	10 (7.2)	10 (7.2)	
	High	44 (31.7)	16 (11.5)	8 (5.8)	20 (14.4)	
	Total^b^	–	74 (53.2)	34 (24.5)	31 (22.3)	

* p-value based on the sign test; ^a^ this column shows the number (%) of students with low, average and high levels of the outcomes before intervention; ^b^ these rows show the number (%) of students with low, average and high levels of the outcomes after intervention

Moreover, for psychological well-being, significant differences were found in the frequency percent of subjects who reported low (34.48% before intervention vs. 26.89% after intervention) and high (65.51% before intervention vs. 73.1% after intervention) levels of psychological well-being (Table 6).

**Table 6:** Psychological well-being before and after intervention in two groups

Variables	Groups	Total^a^	After intervention, n (%)		p-value*
			Low	High	
Psychological well-being Before intervention	Experimental (n = 145, 100%)				
	Low	50 (34.48)	10 (20)	40 (80)	0.00
	High	95 (65.51)	29 (30.52)	66 (69.47)	
	Total^b^	–	39 (26.89)	106 (73.1)	
	Control (n = 139, 100%)				
	Low	51 (36.69)	37 (72.5)	14 (27.45)	0.14
	High	88 (63.3)	24 (27.2)	64 (72.7)	
	Total^b^	–	61 (43.8)	78 (56.11)	

*McNemar test; ^a^ this column shows the number (%) of students with low/high levels of psychological well-being before intervention; ^b^ these rows show the number (%) of students with low/high levels of psychological well-being after intervention

The mean scores comparison of variables before and after the intervention is presented in Table 7. After intervention, statistically significant improvements were only found in life satisfaction, happiness and stress in both the intervention and control groups, but the differences were more significant in the intervention (*p* ≤ 0.001), compared to control (*p* ≤ 0.05) group.

**Table 7:** Mean score comparison of the psychological wellbeing and its associated factors before and after the intervention

	Before intervention	After intervention	Mean Difference	p. value
	Mean (SD)	Mean (SD)		
Self-efficacy				
Experiment	58.57 (10.21)	58.86 (10.35)	0.29	0.67
Control	58.35 (9.87)	59.28 (9.34)	0.93	0.18
Hopefulness				
Experiment	31.77 (3.78)	31.97 (4.01)	0.2	0.51
Control	31.64 (3.98)	32.09 (4.00)	1.55	0.18
Life satisfaction				
Experiment	20.64 (6.10)	22.80 (6.74)	2.16	0.001
Control	21.17 (6.64)	22.52 (6.91)	1.35	0.01
Happiness				
Experiment	1.12 (17.67)	1.18 (19.26)	0.06	0.001
Control	1.14 (20.85)	1.17 (22.24)	0.03	0.04
Stress				
Experiment	21.72 (5.97)	19.43 (6.85)	− 2.29	0.001
Control	21.31 (5.70)	19.64 (6.87)	− 1.67	0.01
Psychological wellbeing				
Experiment	31.46 (16.47)	29.64 (17.69)	− 1.82	0.15
Control	30.29 (13.90)	27.50 (15.73)	− − 2.79	0.11

## Discussion

Our aim in the present study was to determine the effects of a school-based mental health promotion program on some mental health parameters among female high-school students in Tabriz, Iran. The picture of mental health is starting to change in Iran [35], and policymakers are now paying more attention to adolescents and youth, but especially university students [35]. However, a research on 13,486 Iranian students [36] showed that adolescents at the middle schools are having trouble with their mental health. Conducting our school-based intervention that encompassed a variety of physical and mental health components, some changes were seen in the mental health of students. For instance, the proportion of students reporting medium level of happiness increased by about 33.0%, which was higher than the changes observed in all other levels of happiness between the intervention and control groups (Table 5).

Happiness is not only a component of good health [39], but also an indicator of social progress [40]. Because happiness may be considered as a remarkable trait for both people themselves and those around them. Our results on happiness are consistent with those reported in other studies, which showed that by promoting the happiness parameter, mental health [21] and functioning [41] of individuals may be ameliorated. The mechanism by which happiness is ameliorated might be through improved social relations and level of physical activity [1, 11, 14, 17]. For instance, activities such as cooking and listening to music, which were parts of our interventional program, are seen to influence happy times. Similarly, stress management programs, such as ours, are also seen to independently promote happiness [19, 20]. The happiness parameter did not reach significance in our population between the intervention and control groups, which might not be unexpected, due to the possible influences of numerous factors on the propensity towards stress and the numerous stressors that adolescents may remain exposed to on a day-to-day basis [11]. Such stressors may continuously affect the social, emotional and cognitive components that may be involved in “being happy” [35, 42, 43].

Moving further, we also found improvement in life satisfaction of our participants. Life satisfaction among adolescents is important because it may directly impede school performance and success in the future adulthood life. It is a recognized measure of mental well-being [44], and an important outcome in not only healthy state [45] but also diseased conditions, both acute [46] and chronic [47]. Poor life satisfaction is generally a risk indicator [48]. Based on an earlier survey in the Iranian context, life satisfaction decreases as the level of schooling increases from elementary to high school, and those in high school are 0.63 times less likely to be satisfied with their life, compared to elementary school students [35]. The mechanism by which improvement in life satisfaction occurs may encompass a large variety of factors, like having joyful environment [49], access to joyful tools such as music [49], enhanced social communication [50] and improvement in diet and physical activity. These results are similar to ours and also the observations reported in another study, based on which, improvements in life satisfaction through engaging in physical and/or mental activities are more long lasting than changing one’s external circumstances [30]. Besides, using the SWLS scale, we made a systematic evaluation of life satisfaction unlike a single-item scale used in a previous study [51].

In our findings, we also noted a change in psychological well-being besides subjective well-being as above. There is still debate on whether psychological and subjective well-beings are two separate but related dimensions or one overarching construct [52]. Nevertheless, we used the concept of psychological well-being according to Ryff’s definition [53], even though a variety of concepts and measures are present [54]. Longitudinal studies have shown that the high level of psychological well-being, as a protective factor against mental illness and psychopathology [55–57], are associated to biological markers of physical health, risk reduction and longer life-duration [53]. Although others have shown that psychological well-being is stable across time [58], we saw an improvement in our population, which matches with the results of other studies [59, 60]. Since psychological well-being improved in our study, we can deduce that our adolescents might indeed suffer from psychological and/or somatic complaints [52]. We had chosen interventions on the basis of shortages and unmet needs that subjects were reported to have, which matches with the findings of others who showed that a personal approach with face-to-face contact seems to work better than self-help or group interventions [52]. Moreover, since we found an improvement in psychological well-being, we suspect this to be a state-like characteristic rather than trait, as a trait would be very hard to change especially in a short period of time.

Lastly, the participants were recruited from high-schools and may not be a representative of all adolescents in Iran, limiting the external validity of our findings. Moreover, happiness may be considered as a difficult and very personal definition. So, the measurability and/or comparability of happiness should be conducted with caution. We could not analyze many parameters, such as family environment, that seem to affect mental health. After intervention, significant improvements were found in life satisfaction, happiness and stress in both the intervention and control groups, which warns the possible effects of pre-test on the variables. So, it is recommended for future research to consider stronger types of experimental designs, like Solomon Four-Group Design, to control for such threats to internal validity. We had also a short-term follow up, due to time limitations of the research team. Conducting long-term follow ups could provide us with the long-term effects of the program over time. As usual, our results rely on self-reports. In some cases, self-reported well-being measures correlate with social desirability [61]. However, the reliability and validity of more objective ways of measurement such as biological markers or automatic behavioral analyses are yet to be proven.

## Conclusion

Despite the limitations, we concluded that implementing such school-based mental health promotion programs with educational and environmental change and psychophysical strategies may improve some parameters of mental health, including happiness, life satisfaction and psychological well-being among adolescent females. The implementation of SMHPP, as a low-cost, needs-based and multifaceted program, showed promise in promoting mental health among adolescents. Such effectively implemented programs should be supported by health policymakers, particularly in developing countries where there is usually limited resources for school mental health promotion interventions. Our findings also highlighted the importance of ongoing support provision during program implementation. As continual and long-term implementation of such interventions in schools seems to be with difficulties, school health policymakers should focus on school teachers in general and school healthcare providers/nurses in particular, as those who can provide students with ongoing mental health support in the schools. Our work also provided means for reducing burden of poor mental health among adolescents in a non-western cultural context. This is an important evidence for the development and implementation of interventions and policies in the field of mental health promotion.

## Data Availability

The datasets used and/or analyzed during the current study are available from the corresponding author on reasonable request.
